# Ectopia cordis thoracique sporadique: description clinique d'un cas

**Published:** 2012-11-21

**Authors:** Toni Kasole Lubala, Augustin Mulangu Mutombo, Tina Katamea, Nina Lubala, Arthur Ndundula Munkana, Maguy Sangaji Kabuya, Joséphine Kalenga Monga, Oscar Numbi Luboya

**Affiliations:** 1 Faculté de Médecine, Université de Lubumbashi, 1825 Lubumbashi, République Démocratique du Congo; 2 Centre Interdisciplinaire de Génétique au Congo, CIGEC, Lubumbashi, République Démocratique du Congo

**Keywords:** Ectopia cordis, malformation cardiaque, coeur, République Démocratique du Congo, Ectopia cordis, cardiac malformation, heart, Democratic Republic of the Congo

## Abstract

Nous décrivons un cas d'ectopia cordis, une malformation cardiaque congénitale extrêmement rare dans laquelle le coeur est partiellement ou complètement situé en dehors des limites de la cage thoracique. Dans le cas que nous décrivons, elle est thoracique et isolée. Ce cas a été diagnostiqué en salle de naissance au Katanga, au sud de la République Démocratique du Congo. Il s'agit du premier cas documenté chez un nouveau-né Congolais.

## Introduction

L'ectopia cordis est une malformation cardiaque congénitale extrêmement rare dans laquelle le coeur est partiellement ou complètement situé en dehors des limites de la cage thoracique. Sa prévalence est estimée à 5.5 à 7.9 pour un million de naissances vivantes [1]. Quatre types ont été décrits: cervical (5%), thoracique (65%), abdominal (10%) et thoracoabdominale (20%) [2]. La forme thoraco-abdominale est généralement associée à la pentalogie de Cantrell ou une de ses variantes qui inclut un sternum bifide, un défect du diaphragme, de la paroi abdominale antérieure ainsi qu'une malformation intracardiaque [1]. Nous rapportons un cas d'ectopia cordis thoracique isolé. Ce cas a été diagnostiqué en salle de naissance au Katanga, au sud de la République Démocratique du Congo. Il s'agit du premiers cas documenté chez un nouveau-né Congolais.

## Patient et observation

**Figure 1:**
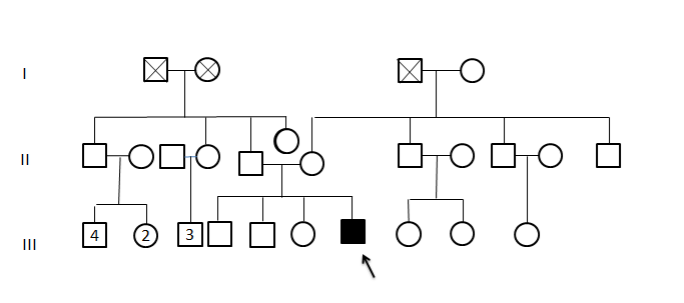
Pédigrée du propositus montrant la nature sporadique de son ectopia cordis

**Figure 2:**
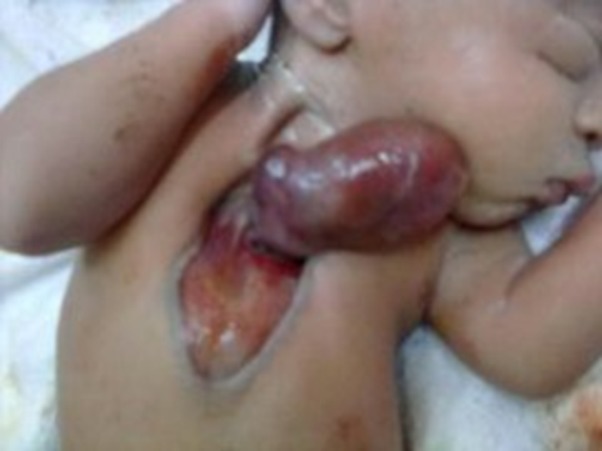
Défect de la paroi thoracique antérieure avec ectopie cardiaque complète

Il s'agit d'un nouveau-né vivant et à terme, de sexe masculin. Il est né par voie basse dans un centre de santé de la ville et référé au service de néonatologie de l'Hôpital Provincial de Référence Jason Sendwe à une heure de vie pour ectopie cardiaque. Il est cadet d'une fratrie de 4. Ses frères et s'urs ne présentent aucune malformation congénitale visible (Figure 1). Son père est âgé de 38 ans et sa mère de 30 ans. Leur mariage n'est pas consanguin. La mère n'a consommé ni alcool, ni tabac pendant la grossesse et aucune infection n'a été rapportée. La grossesse n'a pas été suivie dans le cadre des consultations prénatales. A l'examen clinique, ses mensurations sont normales (Poids 2600g; Taille 49cm; Périmètre crânien: 35 cm); le patient est dyspnéique et présente une cyanose centrale. Il n'y a pas de dysmorphie faciale particulière. On observe un large défect de la paroi thoracique antérieure consécutive à une agénésie du 2/3 inférieur du sternum (Figure 2). A travers ce défect le coeur, macroscopiquement d'aspect normal, fait irruption (Figure 2). L'apex du coeur est orienté en haut, en direction du menton. Le coeur n'est recouvert ni d'une membrane séreuse ni d'un revêtement cutané. Il n'y a pas d'autre malformation externe visible. Le patient est décédé quelques heures après sa naissance, avant que la moindre chirurgie ne soit réalisée. A l'autopsie, on observe une hernie diaphragmatique antérieure. Aucune malformation intracardiaque n'est objectivée.

## Discussion

L'ectopia cordis est une malformation congénitale rare décrite comme une malposition partielle ou complète du coeur à l'extérieur de la cage thoracique. Ce terme a été pour la première fois utilisé par Haller en 1706. Quatre types ont été décrits selon que le coeur fait irruption à travers un défect cervical, thoracique, thoracoabdominal ou abdominal [2]. Dans le cas que nous décrivons, le défect à travers lequel le coeur s'extériorise est situé sur la face antérieure du thorax. Il s'agit donc d'une ectopia cordis thoracique. Chez notre patient comme dans la plupart des cas décrits dans la littérature, la malformation était sporadique et aucune récurrence familiale n'a été rapportée [3].

La majorité des patients décrits dans la littérature avaient des malformations cardiaques associées [3]. Il s'agit généralement de défects du septum inter auriculaire ou inter ventriculaire, voire d'une tétralogie de falot. L'autopsie réalisée chez notre patient n'a révélé aucune malformation intracardiaque associée [3]. S'agissant des malformations extra cardiaques, nous avons observé chez notre premier patient un sternum bifide dans ses 2/3 inférieur ainsi qu'un défect de la partie antérieure du diaphragme [4].

Bien que la majorité des cas d'ectopia cordis soient isolés, des cas d'association avec un encephalocele, une fente palatine ou même des trisomies 18 ont été rapportés. Quelques cas associant une ectopia cordis à une malformation réductionelle d'un membre ont été rapportés dans la littérature. Citons par exemple celui décrit par Chen et al en 2006, qui présentait en plus d'une ectopia cordis, une hypoplasie du membre supérieur droit [5].

La majorité des patients sont mort-nés ou décèdent dans les heures ou jours qui suivent la naissance [1]. Notre patient est décédé à 9 heures de vie. Dans un case report Camerounais, le patient a survécu jusqu'au 7 ième mois de vie en dehors de toute chirurgie [2]. Notons que dans ce cas, le revêtement cutané était intact et qu'il n'y avait aucune autre malformation associée. Dans notre cas, le coeur n'est recouvert ni d'une membrane séreuse ni d'un revêtement cutané. Les données de la littérature révèlent que le coeur est totalement découvert chez 41% des patients. Il est recouvert d'une membrane séreuse chez 31% des patients et d'un revêtement cutané dans 27% des cas [6].

Le pronostic dépend du type d'ectopia cordis, de la capacité de la cage thoracique, de l'instabilité hémodynamique due à la compression des gros vaisseaux, de la présence ou de l'absence d'un revêtement cutané ainsi que de la présence ou l'absence de malformations extracardiaques associées. Dans une autre série de 13 cas présentant une ectopia cordis, 5 patients qui n'avaient aucune malformation extracardiaque ont survécu de 3.5 à 9.5 ans après la correction chirurgicale. Une correction chirurgicale définitive est donc possible [7].

Dans les pays en développement, que le diagnostic soit anténatal ou post-natal, le pronostic est presque toujours fatal. Une étude réalisée sur une série de 10 patients ayant bénéficié d'un diagnostic anténatal a montré une issue uniformément fatale en dehors de toute prise en charge [6]. L'absence de service de chirurgie cardiovasculaire explique en grande partie ce pronostic.

## Conclusion

L'ectopia cordis est une malformation congénitale extrêmement rare ayant presque toujours un pronostic fatal dans les pays à faible revenus.
